# Cardiovascular risk in children and adolescents with end stage renal disease

**DOI:** 10.6061/clinics/2019/e859

**Published:** 2019-06-11

**Authors:** Maria Luiza do Val, Fernanda Souza Menezes, Henrique Tsuha Massaoka, Valeska Tavares Scavarda, Adriano Czapkowski, Heitor Pons Leite, Valdir Ambrósio Moises, Sergio Aron Ajzen, João Tomas de Abreu Carvalhaes, José Osmar Medina Pestana, Paulo Koch‐Nogueira

**Affiliations:** I Departamento de Pediatra, Universidade Federal de Sao Paulo (UNIFESP), Sao Paulo, SP, BR; II Graduacao, Universidade Federal de Sao Paulo (UNIFESP), Sao Paulo, SP, BR; III Departamento de Cardiologia, Universidade Federal de Sao Paulo (UNIFESP), Sao Paulo, SP, BR; IV Departamento de Radiologia, Universidade Federal de Sao Paulo (UNIFESP), Sao Paulo, SP, BR; V Departamento de Nefrologia, Universidade Federal de Sao Paulo (UNIFESP), Sao Paulo, SP, BR

**Keywords:** Chronic Kidney Disease, Cardiovascular Disease, Children, Intima-Media Thickness, Ecocardiography

## Abstract

**OBJECTIVES:**

To evaluate cardiovascular involvement in children and adolescents with End Stage Renal Disease (ESRD) and to characterize the main risk factors associated with this outcome.

**METHODS:**

Cross-sectional study of 69 children and adolescents at renal transplantation and 33 healthy individuals matched by age and gender. The study outcomes were left ventricular mass z-score (LVMZ) and carotid artery intima-media thickness (CIMT). The potential risk factors considered were age, gender, CKD etiology, use of oral vitamin D and calcium-based phosphate binders, systolic and diastolic blood pressure, body mass index z-score, time since diagnosis, dialysis duration, serum levels of ionic calcium, phosphorus, parathyroid hormone, fibroblast growth factor (FGF 23), uric acid, homocysteine, cholesterol, triglycerides, C-reactive protein (CRP), vitamin D and hemoglobin.

**RESULTS:**

In the multivariate analysis, the factors associated with LVMZ were dialysis duration, age, systolic blood pressure, serum hemoglobin and HDL cholesterol levels. Regarding CIMT, in the multivariate analysis, systolic blood pressure was the only factor associated with the outcome.

**CONCLUSION:**

Children exhibited important cardiovascular involvement at the time of the renal transplantation. Both of the studied outcomes were independently associated with systolic blood pressure. For this reason, controlling blood pressure seems to be the main therapy to minimize cardiovascular involvement in children with ESRD.

## INTRODUCTION

Chronic kidney disease (CKD) is a public health concern for adult and pediatric patients 1. In 2010, 2,618 million people in the world underwent renal replacement therapy (RRT) 2, and the prevalence of children undergoing RRT in Brazil was 20 cases per million individuals in this age group in 2012 3.

Cardiovascular disease (CVD) is a leading cause of death in pediatric CKD patients 4-7. In children undergoing dialysis, the mortality associated with cardiac disease is one thousand times higher than in normal children 7. CVD was the main cause of death in patients undergoing dialysis, affecting 33% of cases in a cohort of US children followed from 1995 to 2010 8.

In adults with CKD, CVD results from an interaction of risk factors that are grouped into a) traditional factors, such as hypertension, diabetes, hypercholesterolemia, smoking, sedentary lifestyle, white ethnicity, aging, glucose intolerance, psychosocial stress, family history of heart disease, malnutrition, obesity and male gender; and b) non-traditional factors associated with CKD 9,10. The CKD associated factors are either hemodynamic (volume overload, arteriovenous fistula and anemia) or metabolic, such as oxidative stress; inflammation; hyperhomocysteinemia; proteinuria; increased renin-angiotensin-aldosterone activity; abnormal calcium, phosphorus and vitamin D metabolism; increased serum FGF-23 levels; dyslipidemia; hypoalbuminemia; increased pro-thrombotic factor levels; endothelial dysfunction and infection (*Chlamydia pneumoniae*) 11. When compared to adult patients, children with CKD are less exposed to traditional risk factors 12, which allows for privileged observation of the role of non-traditional factors.

Considering that infarction and stroke are late events not usually experienced in the pediatric age range, we evaluated cardiovascular changes in children and adolescents with CKD at the time of renal transplantation (RT), considering echocardiography and carotid ultrasound as surrogate endpoints, i.e., substitutes for late clinical events. These changes have already been described in children and adolescents in economically developed countries, but reports are scarce in countries in the southern hemisphere.

The present study aimed to evaluate cardiovascular involvement in children and adolescents with ESRD at the time of (RT) using echocardiography and carotid ultrasound. Additionally, we aimed to characterize the main risk factors associated with these outcomes.

## METHODS

We performed a cross-sectional study of 69 patients aged 18 years or less who underwent (RT) at Hospital do Rim-UNIFESP. A control group of healthy children paired by sex and age who were treated in the adolescents outpatient service from the same institution was also included. The data were collected from March 2012 to December 2014.

The study was approved by the Ethics and Research Committee of the Federal University of São Paulo (Protocol 2031/11). All patients enrolled in the study signed a consent form.

The exclusion criteria were: congenital or structural cardiac abnormalities and primary myocardial disease; children with active infectious disease, characterized by fever and bacteremia, or under antimicrobial treatment; diabetes, active inflammatory diseases (e.g., vasculitis and systemic lupus erythematosus), or genetic or endocrine diseases with disorders in calcium or phosphorus metabolism; smoking (in adolescents); and the presence of a venous catheter near the carotids.

Left ventricular mass z-score (LVMZ) measured by echocardiography and carotid artery intima-media thickness (CIMT) measured by carotid ultrasound in both groups were expressed as a continuous quantitative variable.

The following risk factors were considered: age at transplantation, gender, body mass index z-score, time (months) since CKD diagnosis, duration (months) of dialysis before the renal transplantation, etiology of the CKD (undetermined, urinary tract malformation, glomerulopathies, other diagnoses), systolic blood pressure (SBP), diastolic blood pressure, serum hemoglobin levels, serum albumin levels, serum total cholesterol levels, serum HDL cholesterol levels, serum triglycerides levels, C-reactive protein (CRP), uric acid, homocysteine, serum ionic calcium levels, serum phosphorus levels, serum calcium x phosphorus product levels (CaxP product), serum parathyroid hormone (PTH) levels, serum fibroblast growth factor (FGF23) levels, serum vitamin D levels, vitamin D use and use of calcium-based phosphate binders (categorically evaluated as yes or no, with no confirmation of the time of use or adherence).

To prevent the requirement of invasive and painful procedures in healthy individuals, blood sampling was not performed in the control group.

### Evaluation of the Left Ventricular Mass Z-Score Outcome

After a median follow-up of 24 days (IQR=16 to 30) following RT transthoracic color Doppler echocardiography was performed by 1 of 2 expert physicians using a standard VIVID 7 dimension device (General Electric® Healthcare) with a 5-mHz transducer. To calculate the left ventricular mass Z-scores (LVMZ), Parameter Z software was used according to the American Society of Echo's Guidelines and Standards for Performance of a Pediatric Echocardiogram 13.

We used American Society of Echocardiography guidelines (iASE version 3.0.4) to evaluate the left ventricular mass index (LVMI), including relative wall thickness and left ventricular geometry. Diastolic dysfunction was evaluated with tissue Doppler and expressed as a binary variable (present or absent diastolic dysfunction) 14.

### Evaluation of Carotid Artery Intima-Media Thickness

All ultrasound exams were performed by the same examiner using a 3-12-MHz multi-frequency linear transducer device (LOGIQ 7, General Electric Health Care®). The patients were maintained in slight neck hyperextension with the chin slightly rotated laterally in the opposite direction to the transducer after at least ten minutes of rest 15.

The CIMT measure was defined as the distance between the edges of the lumen-intimal interface and the medial-adventitial interface of the distal wall, measured bilaterally in the common carotid artery 1 cm below the bifurcation 16. Two measures were manually performed with the caliper method on each artery scan and the mean of the results was calculated.

For the control group, the echocardiogram and carotid ultrasound were obtained using the same methodology as for the study group.

### Statistical Analysis

The data were expressed as the mean and standard deviation or median and interquartile range (IQR) according to the distribution of the variables. To compare the two groups, we used Student's t-test for quantitative variables and the chi-squared test or Fisher's exact test for proportions.

To evaluate a possible association between risk factors and outcomes (left ventricular mass z-score and carotid artery intima-medial thickness), a simple linear regression model was used for each outcome. Then, variables with *p*<0.10 in the simple linear regression were selected for inclusion in a multivariate linear regression model. In the multivariable models we included up to 5 potential risk variables to respect the proportion of introducing one risk variable for each 10 to 15 individuals in the sample. The covariables that did not exhibit a significant association with the studied outcome were removed one by one (i.e., the backward selection method). Lastly, the interaction terms between the variables that remained in the final models were investigated.

For all of the tests, a limit of *p*<0.05 was adopted to reject the null hypothesis, and all calculations were performed using Stata software 14.2® (College Station, TX77845, USA).

The study was approved by the Ethics and Research Committee of the Federal University of São Paulo (Protocol 2031/11). All patients enrolled in the study signed a consent form.

## RESULTS

During the data collection, there were 79 pediatric transplants, with all the recipients being candidates for participation in the study. Two refused to participate and eight were excluded, four for infections and four because they did not attend the hospital for the examinations.

The demographic, anthropometric and clinical data of the study sample are presented in Table 1. There were no significant differences regarding age or sex between the groups, whereas the weight, height and BMI z-score were significantly lower in ESRD children.

Regarding the etiology of CKD, there were 26 cases of urinary tract malformations (38%), 16 cases of glomerulopathies (23%), 23 cases of undetermined diseases (33%) and four children with other diagnoses (6%).

The median time span since the CKD diagnosis to RT was 35 months (IQR=13–72), and the median duration of RRT was 14 months (IQR=8–23). In 7/69 cases (10%), a preemptive RT was performed. Among the remaining 62 patients, the dialysis methods were exclusive hemodialysis (HD) in 35 patients (51%), exclusive peritoneal dialysis (PD) in 14 cases (20%), and both HD and DP in 13 (19%) cases. Regarding the type of RT, 65 children (94%) received a deceased-donor kidney.

The blood pressure values were higher among ESRD children when compared to controls. Among cases, 44 patients (64%) used antihypertensive drugs and 16 had stage 1 hypertension 17, 9 had stage 2, 5 were pre-hypertensive, and 14 had normal blood pressure. In the group of patients not using antihypertensive drugs, 4 had high blood pressure levels: two with stage 1 hypertension, one with stage 2, and one with pre-hypertension. None of the individuals from the control group required hypertension medication, and all of them had normal blood pressure.

### Echocardiographic Parameters

**Figure 1:**
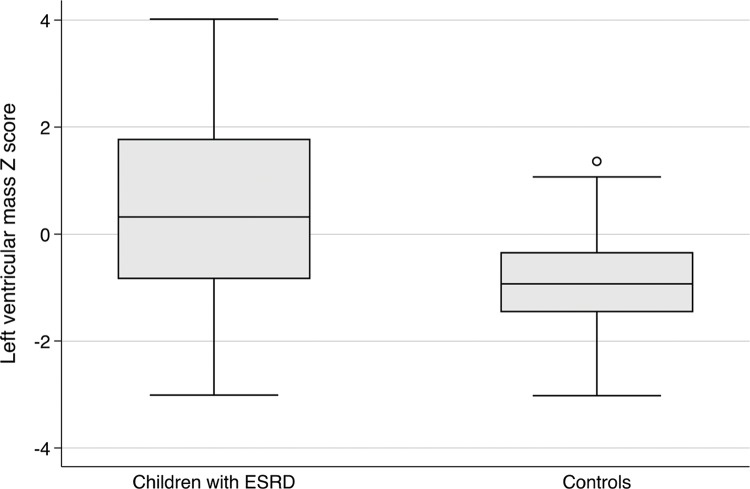
Left ventricular Z-score according to study group.

The left ventricular mass z-score (LVMZ) was 0.48 (SD=1.75) in CKD patients and -0.94 (SD=1.00) in patients from the control group, displaying a significant difference between groups (Figure 1). The LVMZ was increased (>2) in 14 patients among cases (20%) and in no individuals from the control group.

Among patients with CKD, 42 children (60.9%) exhibited normal left ventricular geometry, whereas concentric hypertrophy was observed in 14 patients (20.3%), eccentric hypertrophy in 11 patients (15.9%) and concentric remodeling in two other (2.9%). Conversely, all individuals from control group had normal left ventricular geometry (*p*<0.001).

The left ventricular systolic function was preserved in all of the patients, except for one case that exhibited moderate dysfunction. All of the controls had normal function. The analysis of the diastolic function was not possible in one case due to tachycardia. Nine patients with CKD (13%) exhibited altered diastolic dysfunction (13%), whereas controls were normal (*p*<0.001).

The results of the univariate and multivariate linear regression are compiled in Table 2.

In this model, among all the nine pre-selected covariables, we opted to exclude four in order to comply with the proportion of including one counfounder for each ten to fifteen individuals in the sample. Hence, we excluded DBP because it was strongly correlated with SBP and also etiology of the CKD, serum FGF23 and vitamin D use because these were the variables with less previously published evidence to explain changes in left ventricular mass. All the selected five factors showed a significant association with LVMZ in this model: dialysis duration, age at transplantation, SBP, serum hemoglobin levels and serum HDL levels. The details of these associations are as follows: for each 1-month increase in dialysis duration, a 0.017 SDS increase in LVMZ is observed; for each additional year of age at RT, a 0.08 SDS increase in LVMZ is expected; each 1 mmHg increase in SBP is associated with a 0.024 SDS increase in LVMZ; for each 1 g/dL increase in serum hemoglobin, a 0.20 SDS decrease in LVMZ is expected; and for each 1 mg/dL increase in HDL, a decrease of 0.04 SDS in LVMZ is predicted. The analysis of the interaction between these variables did not show significant effects, suggesting that these associations with LVMZ are independent.

### Analysis of the Carotid Artery Intima-Medial Thickness

**Figure 2:**
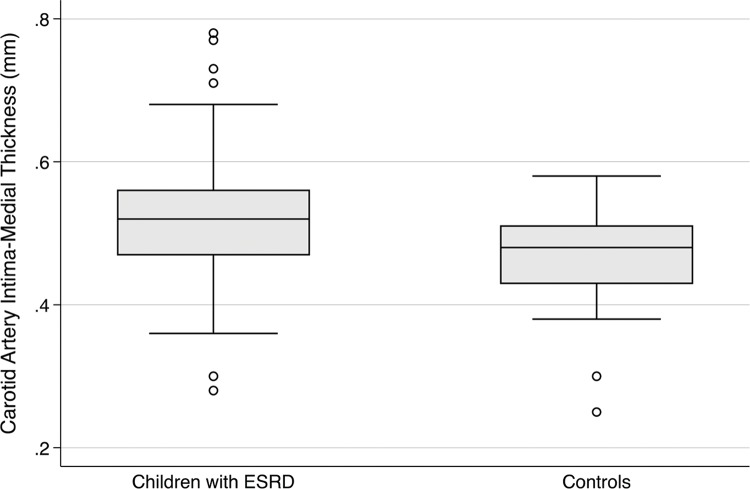
Carotid artery intima-medial thickness according to study group.

When comparing the carotid artery intima-medial thickness (CIMT), we found a significant difference between the groups, with a greater thickness in patients with ESRD (0.52±0.10 mm versus 0.46±0.07 mm, *p*=0.028). This result demonstrates arterial involvement in children with ESRD, as illustrated in Figure 2. The linear regression analyses for this outcome are compiled in Table 3.

Age, SBP, diastolic blood pressure (DBP) and vitamin D use exhibited an association with CIMT and were included in the multivariate linear regression model, after which there was only an association with SBP. According to this analysis, each 10-mmHg increase in SBP was associated with an increase of 0.015 mm in CIMT.

## DISCUSSION

The main finding of the present study was that ESRD children and adolescents exhibited cardiovascular involvement at the time of RT presenting increased LVM and CIMT when compared to controls. Such outcomes are substitutes for late clinical events at the pediatric age range. However, these outcomes are relevant because they are recognized risk factors for predictable clinical outcomes, especially in adults 18,19.

Cardiac abnormalities are frequent in adults and children with CKD and contribute to morbidity 7,9,20. In adults, a prospective study evaluating 161 patients undergoing HD showed that each 1 g/m2.7 increase in LVMI was associated with a 62% increase in the risk of adverse cardiovascular events. This association suggests that changes in LVM have independent prognostic value for cardiovascular events, reinforcing echocardiography as a tool for monitoring cardiovascular risk in patients undergoing dialysis 21.

Left ventricular hypertrophy (LVH) is common in pediatric and adult patients undergoing dialysis 9. This change may occur early and has a prevalence ranging from 30% in mild to moderate CKD 22 to 73% in children undergoing dialysis 23-26. There are different methods for indexing LVM, a topic that is widely discussed in the literature 22,25,27. In this research, considering that LVM is influenced by the age and size of pediatric patient, we opted to use the LVM z score to express the magnitude of the deviation from the mean 28. In study involving children in different stages of CKD, the use of the z-score allowed for the identification a higher LVH proportion in the dialysis group when compared to other criteria, such as LVM indexed to body surface area 27.

The first variable that exhibited an independent association with LVMZ was age at RT, which is biologically plausible. However, because we use the z score to express the LVM, the effect of age on any body size measurement was attenuated. Therefore, this association is likely related to CKD and not simply due to LVM increase with age. In addition to age, we observed the effect of dialysis duration on LVMZ and it is possible that the association between LVM and age is somehow related to dialysis duration. In contrast to our findings, the association between LVM and dialysis duration was not found in another study involving 64 patients undergoing dialysis 29. However this study was retrospective, and we believe that our finding highlights the importance of limiting dialysis duration and encouraging preemptive RT to decrease the frequency of LVH.

Another finding was the association between serum hemoglobin and LVM, which is consistent with data from the literature that reports an association between anemia and LVMI 22,30. In a cross-sectional study evaluating 156 children undergoing dialysis with similar mean age to that of our study, an independent association between low serum hemoglobin and LVMI was reported 22. In agreement to these findings, a more recent study on 46 patients undergoing dialysis also showed anemia and hypertension as predictive factors of LVMI in patients 5 to 21 years of age 30.

Amongst the potential risk factors tested in the present study, only SBP exhibited an association with both of the studied outcomes. This finding agrees with most studies in the literature 22,24,31,32 and has implications for daily clinical practice, reinforcing the need for strict control of this parameter in CKD patients. Specifically in children undergoing dialysis, an association between hypertension and LVH was reported in another study on Brazilian children 33. Moreover, if we consider that hypertension is common in both the short- 34 and long term 35 following RT in children, adequate diagnosis and treatment of this complication has increased importance to reduce the risk for cardiovascular complications in all stages of CKD. It is worth noting that data from another cross-sectional study showed an association between SBP and LVMI even in non-hypertensive CKD patients, suggesting that the target in current recommendations for controlling the SBP must be reconsidered 24.

Low serum HDL is associated with increased coronary disease in adults with CKD, and lower baseline concentrations when compared to the general population have been reported. These lipid abnormalities may produce a 1.2-1.4-fold higher risk for coronary disease 36. Our findings confirm the possibility that decreasing serum HDL cholesterol in children increases LVM, which may have implications for the clinical treatment of these patients.

Previous studies have reported normal CIMT values in children from the northern hemisphere 16,37 as well as in Brazil 38. However, such studies are scarce and not internationally validated. For this reason, we chose not to calculate the CIMT z-score and our analyses were performed with the absolute value of this measure. In a study that evaluated patients aged between 10 and 20 years of age without cardiovascular disease, associations between CIMT and age and also between body size and blood pressure were observed in healthy adolescents 16. Similarly, in a European multicenter study, a significant relationship was found between the SBP z-score and BMI as independent positive predictors associated with CIMT 37.

In a study evaluating 101 children aged between 2 and 18 years of age and healthy controls, dyslipidemia and hypertension were associated with an increase of in the CIMT, 39. We also found a significant association between SBP with CIMT, and there was no association with calcium, phosphorus, gender or CKD etiology, which is similar to the findings of Litwin et al. 12.

The results of the present study must be interpreted considering its limitations. The first is the cross-sectional design of the study, which prevents the establishment of causal relationships between risk factors and the studied outcomes. The development of cardiovascular complications is a process that occurs over time, and cross-sectional studies, only one time point in this progression can be evaluated. Another study feature that prevented us from formulating more in-depth analyses was the lack of data on both dialysis quality and the duration of the medications that the patients were receiving. The fact that patients of the study had been referred for from different regions of the country precluded access to their medical records in the dialysis clinics of origin, rendering it impossible to obtain data on prescription duration, changes in dosages and adherence. This factor limited our analysis of the role of dialysis quality and the use of calcium-based phosphate binders, vitamin D analogs and antihypertensive medications.

Nevertheless we believe that our findings indicate that there is significant cardiovascular involvement in at least 1/5 of pediatric ESRD patients at the time of RT. We concluded that children with CKD show cardiac involvement and that the control of modifiable risk factors as hypertension and anemia must be considered therapeutic aims. The independent association between SBP and both markers suggests an opportunity for interventions that aim to prevent cardiovascular complications.

## AUTHOR CONTRIBUITIONS

Do Val ML was responsible for the preparation of the project, participated in all planning, responsible for selection, recruitment, patient data collection and double database typing, execution and manuscript writing. Menezes FS was responsible for selection, recruitment, patient data collection and double database typing and final approval of the manuscript version to be published. Massaoka HT, student of medical graduation, participated in the study conception and was responsible for the graphical elaboration and tabulation of the database. Scavarda VT, pediatric cardiologist, responsible for performing the echocardiogram examinations of the patients and final approval of the manuscript version to be published. Moisés VA provided substantial contributions to conception and design, and analysis and interpretation of cardiologic data. Czapkowski A, radiologist, responsible for performing the ultrasound examinations to measure the patients’ mean intimal complex of the carotid artery and final approval of the manuscript version to be published. Ajzen SA was responsible for the study conception, design, analysis and interpretation of radiologic data, and final approval of the manuscript version to be published. Pestana JO was responsible for data discussion. Leite HP was responsible for data discussion, project supervision and manuscript drafiting and manuscript revising for critically important intelectuall content. Carvalhaes JT, co-supervisor, was responsible for the project revision and final approval of the manuscript version to be published. Koch-Nogueira P, supervisor, participated in all planning, project manager, data analysis and final approval of the manuscript version to be published.

## Figures and Tables

**Table 1:** Demographic, anthropometric and clinical data of the sample according to the study group.

Variable	Cases (n=69)	Controls (n=33)	*p*
Mean Age in Years (SD)	13.1 (4.6)	13.0 (3.7)	0.967
Gender (Male/Female)	39/30	17/16	0.635
Mean Weight in kg (SD)	38.4 (17.1)	47.6 (14.3)	**0.009**
Mean Height in cm (SD)	141.7 (25.6)	152.1 (18.5)	**0.042**
H/A Z-Score (SD)	‐1.6 (1.5)	‐0.1 (0.9)	**<0.001**
BMI Z-Score (SD)	‐0.6 (1.5)	0.3 (0.8)	**0.001**
Pubescent/Pre‐pubescent	40/17	25/8	0.568
Months Since Diagnosis (IQR)	35 (13 to 72)	NA	NA
Months Undergoing Dialysis (IQR)	14 (8 to 23)	NA	NA
Urinary Tract Malformation (n/total)	26/69	NA	NA
Systolic Blood Pressure (mmHg)	122 (21)	99 (11)	**<0.001**
Diastolic Blood Pressure (mmHg)	76 (15)	59 (9)	**<0.001**

SD‐Standard deviation of the mean, H/A‐Height/Age, BMI-Body mass index, NA-Not applicable, IQR‐Interquartile range of the median.

**Table 2:** Univariate and multivariate linear regression analysis of risk factors associated with left ventricular mass Z-score.

Variable	Coefficient	CI	*p*	Coefficient	CI	*p*
Age	0.084	0.013 to 0.155	**0.020**	0.083	0.009 to 0.157	**0.028**
Gender	‐0.334	‐1.008 to 0.339	0.328			
Body Mass Index (BMI)	‐0.055	‐0.362 to 0.251	0.720			
Time Since Diagnosis	0.000	‐0.008 to 0.008	0.991			
Dialysis Duration	0.017	‐0.002 to 0.037	**0.082**	0.017	0.002 to 0.031	**0.020**
Urinary Tract malformation	‐0.755	‐1.562 to 0.050	**0.066**	**NS**		
Systolic Blood Pressure	0.038	0.024 to 0.051	**0.000**	0.024	0.005 to 0.043	**0.011**
Diastolic Blood Pressure	0.045	0.025 to 0.065	**0.000**	**NS**		
Serum Hemoglobin (g/dL)	‐0.214	‐0.416 to ‐0.013	**0.037**	‐0.204	‐0.379 to ‐0.028	**0.023**
Serum Albumin (g/dL)	0.310	‐0.255 to 0.876	0.277			
Serum Cholesterol (mg/dL)	‐0.002	‐0.011 to 0.006	0.555			
Serum HDL (mg/dL)	‐0.048	‐0.086 to ‐0.011	**0.012**	‐0.040	‐0.075 to ‐0.005	**0.026**
Serum TG (mg/dL))	0.002	‐0.002 to 0.006	0.377			
Serum CRP (mg/dL)	0.157	‐0.234 to 0.549	0.425			
Serum Uric Acid (mg/dL)	‐0.064	‐0.289 to 0.160	0.570			
Serum Homoc (μmol/dL)	0.011	‐0.033 to 0.568	0.613			
Serum Ionic Calcium(mg/dL)	‐4.065	‐8.990 to 0.859	0.104			
Serum Phosphorus (mg/dL)	0.166	‐0.101 to 0.434	0.219			
CaxP Product (mg/dL)	0.010	‐0.019 to 0.040	0.475			
Serum PTH (pg/mL)	0.000	‐0.000 to 0.001	0.145			
Serum FGF23 (pg/mL)	0.000	0.000 to 0.000	**0.038**	**NS**		
Serum Vitamin D (ng/dL)	0.011	‐0.021 to 0.043	0.500			
Vitamin D Use	0.806	‐1.674 to 0.062	**0.068**	**NS**		
Use of Phosphate Binders	‐0.130	‐1.106 to 0.845	0.790			

**Table 3:** Univariate and multivariate analysis of risk factors associated with left carotid artery intima-medial thickness.

Variable	Coefficient	CI	*p*	Coefficient	CI	*p*
Age	0.005	0.001 to 0.010	**0.013**	0.003	‐0.001 to 0.008	0.122
Gender	‐0.013	‐0.050 to 0.023	0.478			
Body Mass Index (BMI)	‐0.008	‐0.021 to 0.003	0.168			
Time Since Diagnosis	‐0.000	‐0.000 to 0.000	0.559			
Dialysis Duration	‐0.000	‐0.001 to 0.000	0.733			
Urinary Tract Malformation	‐0.021	‐0.066 to 0.024	0.364			
Systolic Blood Pressure	0.001	0.000 to 0.002	**0.001**	0.001	0.000 to 0.002	**0.009**
Diastolic Blood Pressure	0.001	0.000 to 0.003	**0.001**	0.000	‐0.001 to 0.003	0.532
Serum Hemoglobin (g/dL)	‐0.005	‐0.016 to 0.004	0.251			
Serum Albumin (g/dL)	0.022	‐0.006 to 0.050	0.122			
Serum Cholesterol (mg/dL)	‐0.000	‐0.000 to 0.000	0.176			
Serum HDL (mg/dL)	0.001	‐0.000 to 0.003	0.181			
Serum TG (mg/dL)	‐0.000	‐0.000 to 0.000	0.181			
Serum CRP (mg/dL)	‐0.004	‐0.020 to 0.010	0.535			
Serum Uric Acid (mg/dL)	0.001	‐0.013 to 0.015	0.881			
Serum Homoc (μmol/dL)	0.002	‐0.001 to 0.006	0.251			
Serum Ionic Calcium (mg/dL)	0.078	‐0.134 to 0.291	0.467			
Serum Phosphorus(mg/dL)	‐0.006	‐0.023 to 0.010	0.450			
CaxP Product (mg/dL)	‐0.000	‐0.002 to 0.001	0.649			
Serum PTH (pg/mL)	‐0.000	‐0.000 to 0.000	‐0.500			
Serum FGF23(pg/mL)	‐0.000	‐0.000 to 0.000	0.684			
Serum Vitamin D (ng/mL)	‐0.000	‐0.001 to 0.001	0.924			
Vitamin D Use	‐0.058	‐0.103 to ‐0.013	**0.011**	‐0.031	‐0.081 to 0.019	0.221
Use of Phosphate Binders	0.024	‐0.025 to 0.074	0.333			
